# The Influence of Surgical Staff Behavior on Air Quality in a Conventionally Ventilated Operating Theatre during a Simulated Arthroplasty: A Case Study at the University Hospital of Parma

**DOI:** 10.3390/ijerph17020452

**Published:** 2020-01-10

**Authors:** Cesira Pasquarella, Carla Balocco, Maria Eugenia Colucci, Elisa Saccani, Samuel Paroni, Lara Albertini, Pietro Vitali, Roberto Albertini

**Affiliations:** 1 Department of Medicine and Surgery, University of Parma, via Volturno, 39, 43125 Parma, Italy; mariaeugenia.colucci@unipr.it (M.E.C.); elisasaccani@libero.it (E.S.); paroni.samuel@gmail.com (S.P.); roberto.albertini@unipr.it (R.A.); 2 Department of Industrial Engineering, University of Florence, via S. Marta 3, 50139 Firenze, Italy; carla.balocco@unifi.it; 3 Freelance Architect, 43126 Parma, Italy; lara.albertini@outlook.com; 4 Hygiene Unit, University Hospital of Parma, Parma, via Gramsci 14, 43126 Parma, Italy; pvitali@ao.pr.it; 5 Clinical Immunology Unit, University Hospital of Parma, Parma, via Gramsci 14, 43126 Parma, Italy

**Keywords:** operating theatre, hip arthroplasty, indoor air quality, biological monitoring, particle counting, microclimatic monitoring, surgical staff behavior

## Abstract

Surgical staff behavior in operating theatres is one of the factors associated with indoor air quality and surgical site infection risk. The aim of this study was to apply an approach including microbiological, particle, and microclimate parameters during two simulated surgical hip arthroplasties to evaluate the influence of staff behavior on indoor air quality. During the first hip arthroplasty, the surgical team behaved correctly, but in the second operation, behavioral recommendations were not respected. Microbiological contamination was evaluated by active and passive methods. The air velocity, humidity, temperature, and CO_2_ concentration were also monitored. The highest levels of microbial and particle contamination, as well as the highest variation in the microclimate parameter, were recorded during the surgical operation where the surgical team behaved “incorrectly”. Turbulent air flow ventilation systems appeared more efficient than in the past and very low air microbial contamination was reached when behavior was correct. Therefore, adherence to behavioral recommendations in operating theatres is essential to not undermine the effectiveness of the heating, ventilation, and air conditioning systems and employed resources.

## 1. Introduction

Surgical site infections (SSIs) following total joint replacement surgery make up the most feared complication, presenting a significant burden in terms of patient morbidity and additional related costs [1]. Many factors increase the risk of SSIs, including patient-related, procedural-related, and management-related factors [1]. In particular, in order to preserve the indoor air quality provided by heating, ventilation, and air conditioning (HVAC), reducing operator movement in the operating theatres during surgical activity is recommended, e.g., keeping operating theatre doors closed, except as needed for passage of equipment, personnel, and the patient, limiting the number of personnel entering the operating theatre to those necessary, and minimize personnel traffic during operations [1,2,3,4]. Microbial contamination of the surgical site is a necessary precursor of SSIs and the air in operating theatres (OTs) represents an important vehicle for SSI-related microorganisms which can fall directly into the wound or land on exposed surfaces and subsequently be transferred into the wound [5]. A Medical Research Council study found a significant correlation between microbial air contamination, wash-out bacterial count, and the incidence of SSIs [6,7]. Therefore, the use of ultra-clean, ventilated OTs with unidirectional airflow was recommended in orthopedic implant surgeries, with maximum air microbial contamination values during operation of 10 colony-forming units per cubic meter (CFU/m^3^) when measured by active sampling [8,9,10], and 350 CFU/m^3^/h [11] and 2 CFU/9-cm-plate/h when measured by passive sampling [12,13,14]. As for particle contamination, an ISO 5 class, i.e., below 3520 particles ≥ 0.5 µm/m^3^, is recommended for hip arthroplasty surgery operating theatres [4,10,15,16]. However, in 2008, a retrospective study unexpectedly showed significantly higher SSI rates after hip prosthesis implantation when using unidirectional airflow ventilation compared with turbulent (mixing) ventilation [17], and a subsequent meta-analysis [18] performed within the framework of developing World Health Organization (WHO) global guidelines [1] for the prevention of surgical site infections showed no difference in risk between unidirectional airflow ventilation and turbulent ventilation for hip SSIs following total hip arthroplasty [18]. On the basis of this meta-analysis, WHO guidelines suggest that unidirectional airflow ventilation systems “should not be used to reduce the risk of SSI for patients undergoing total arthroplasty surgery”, even though “the strength of this recommendation was considered to be conditional, considering the very low quality of the supporting evidence” [1]. Several criticisms were aimed at the studies included in the meta-analysis [19,20,21]; in particular, none of them contained an assessment of air microbial contamination, or took into consideration that, despite unidirectional airflow ventilation, microbial air contamination could still have exceeded recommended threshold values, thereby reducing the effectiveness of the ventilation system.

Different studies showed the strong influence of the amount of people present in the OT and their movements and the influence of doors opening and closing on microbial contamination, air pressure, and microclimatic conditions. Furthermore, surgical staff behavior has a strong impact on indoor air quality and ventilation effectiveness in OTs equipped with HVAC systems with unidirectional turbulent flow [22,23,24,25,26,27,28,29,30,31,32,33,34,35]. In particular, the ISChIA study [22,36] showed a correlation between the number of times the door was opened, the number of people in the OT, and microbial air contamination, highlighting a high level of contamination that exceeded the current threshold values in OTs supplied with unidirectional airflow, which was even higher than the microbial contamination values obtained in conventionally ventilated OTs in some case4s. Other studies showed that in currently used, conventionally ventilated OTs, it was possible to obtain microbial contamination levels lower than the HTM 03-01 recommended values of ≤180 CFU/m^3^ during surgical activity [22,29,37]. However, during bad OT management, these values could also very much exceed the recommended levels [22,29]. From this background research, the aim of the present study was to evaluate microbiological and particle air contamination and microclimate parameters in order to highlight the influence of incorrect behavior on air quality in a conventionally ventilated OT during hip arthroplasties and to compare the obtained results with current standards.

## 2. Materials and Methods

### 2.1. Setting

**Figure 1:**
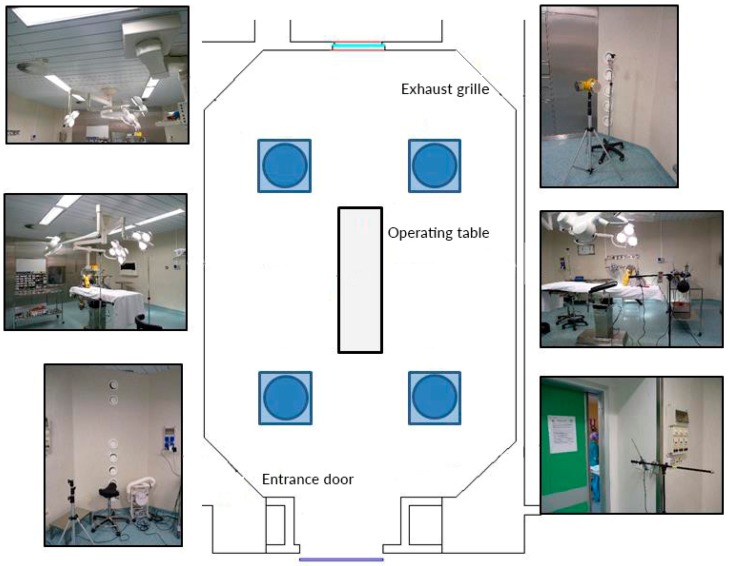
Diagram of the operating theatre showing the sampling points at the operating table, exhaust grille, and the entrance door.

The study was carried out in July 2016 and performed in an ISO 5 [16], conventionally ventilated operating theatre (OT) at the University Hospital of Parma. The OT had a surface area of 42.9 m^2^ and a volume of 123.6 m^3^. It was equipped with two groups of 7 conical outgoing grilles (cross-section of 0.0124 m^2^ each) located near the floor and the ceiling (Figure 1).

Four diffusers were located on the ceiling, each supplying ventilation to one quarter of the room. The annular surface for fluid inlet was 0.26 m^2^ for each diffuser, which were equipped with tilted bladed systems to generate a swirling incoming flow in the room. The HVAC system was equipped with high-efficiency particulate air filters, which could remove particles of ≥0.3 µm with an efficiency of 99.97% and provided 15 air changes per hour. The operating theatre was equipped with an operating table and a lighting system consisting of three joined arms, each one holding three light lamps. A sliding door connected the OT to the corridor.

### 2.2. Monitoring Programme

Measurements of biological and particle air contamination and microclimatic parameters were carried out during two simulated hip arthroplasties under different conditions regarding surgical team behavior, i.e., the number of people present in the OT, the number of times that a door was opened, the movements inside the OT, and conversation between members of the surgical team. The surgical team consisted of three surgeons and one scrub nurse at the operating table on which the patient lay, along with one anesthetist and one nurse circulating inside. The surgical operational conditions, defined as “correct (C)”, corresponded to the surgical operation performed with the surgical team behaving correctly, where the door was only opened three times, the surgical staff’s (six people) movements were reduced to a minimum, and talking was strictly related to the surgical operation. The “not correct (NC)” conditions corresponded to the hip arthroplasty performed while the surgical team behaved incorrectly. Fundamental disturbance factors were simulated and carried out over time, such as the sliding door opening 25 times for ingoing and outgoing operators, the presence of people not part of the surgical team, and unnecessary movement and talking. Each surgical operation lasted 40 min. An interval of 30 min separated the two simulated surgeries. The HVAC system worked throughout both tests and measurements were carried out between the 5th and the 35th minute during the C and NC conditions. During the “at rest (R)” condition (i.e., room complete with all services functioning and equipment installed and operating, but without surgical or healthcare staff or the patient being present), measurements were taken for 30 min as control data. Figure 1 shows the diagram of the operating theatre and the sampling points. Air sampling was performed once in the corridor during routine operational conditions.

### 2.3. Sampling Points

The sampling points were chosen with regard to the importance of not disturbing surgical activity. As shown in Figure 1, microbial air sampling in the operating theatre was performed at the operating table, the exhaust grille, and the entrance door. In the corridor, the air was sampled at a point in front of the OT entrance door. Particle sampling was performed at the operating table. Air temperature and relative humidity were measured at the operating table, exhaust grille, and entrance door, air velocity was measured at the operating table and entrance door, and the mean radiant temperature and CO_2_ were measured at the operating table.

### 2.4. Environmental Monitoring

#### 2.4.1. Microbial Air Sampling

Microbial air sampling was carried out by active sampling to measure the concentration of microorganisms in the air, and by passive sampling to measure the rate at which the microorganisms settled on the surfaces [38,39]. Active sampling was carried out using three DUO SAS Super 360 samplers (International PBI, Milan, Italy) equipped with RODAC plates (5.5 cm in diameter). During the operation, 250 L of air was aspirated 4 times for a total of 1000 L; 1000 L at once was aspirated during the R condition. The flow rate was 180 L per minute (L/min). The samplers were placed at a height of 1 m above the floor. Results were adjusted according to the table provided by the manufacturer and were expressed as CFU/m^3^. Passive sampling was carried out using 9-cm Petri dishes which were exposed to the air for 30 min at 1 m above the floor to determine the Index of Microbial Air contamination (IMA) [13] after one hour.

Triptic Soy Agar (TSA) was used for the total bacterial count and Sabouraud Dextrose Agar (SDA) with chloramphenicol was used for the fungal count. After sampling, the TSA plates were incubated at 36 ± 1 °C for 48 h and the SDA plates at 22 ± 1 °C for 120 h. Microscopic fungi identification was performed via the scotch test and lactophenol blue staining.

#### 2.4.2. Particle Counting

Airborne particles with a diameter of ≥0.5 μm were counted with a laser particle counter Climet CI 754 (Climet Instruments Company, Redlands, CA, USA), certified, and validated in accordance with the correct requirements [16,40]. The suction volume was 75 L/min. Measurements were carried out in triplicate with a start-up delay of 1 min, and a delay time of 5 s between the three suctions of 350 L each.

#### 2.4.3. Microclimatic Monitoring

Microclimatic monitoring was performed using the data-logger LSI LASTEM ELR510M. The instrumental apparatus consisted of three thermohygrometers to measure temperature and humidity, one globe thermometer to measure the mean radiant temperature, one detector to assess the CO_2_ concentration, and two hot wire anemometers for to measure the air velocity. The technical characteristics of the apparatus complied with the specific requirements [41]. Dedicated software was used to set the parameters and record the data.

Measurements were recorded during each of the conditions investigated, i.e., R, C, and NC. The time-step chosen for sample collection was one second, and data processing was set at one minute. In particular, the microclimatic parameters values were measured continuously and acquired every second.

The analyses of the air flow field, temperature distribution map, relative humidity, and CO_2_ distribution for each of the OT conditions (R, C, and NC) considered parameter variations every second. The evaluations, verifications, and comparative analyses of these same parameters for each condition (R, C, and NC) were obtained from the average values taken every minute. For the data post-processing samples, the standard deviation was computed using Bessel’s correction [42]. All of the microclimatic data were analyzed and post-processed from the whole time of the hip arthroplasty, i.e., 30 min worth of acquired data.

## 3. Results

### 3.1. Biological Sampling

Table 1 shows the microbial contamination values obtained at the different sampling points, i.e., at the rest condition and during the two simulated hip arthroplasties.

No fungi were isolated during the R condition active or passive sampling. During surgical activity, the microbial contamination values increased at all sampling points. During the operation performed with the surgical team behaving correctly, the CFU/m^3^ increased 6.5 times, 7.7 times, and 7.2 times at the operating table, HVAC exhaust grille, and entrance door of the OT, respectively, while the IMA values increased from 0 to 2, from 0 to 6, and from 2 to 4, respectively. The lowest values were obtained at the operating table using both CFU/m^3^ and IMA (13 CFU/m^3^ and 2 IMA); the maximum CFU/m^3^ value (29 CFU/m^3^) was recorded at the entrance door of the OT, while the maximum IMA value (6 IMA) was recorded at the exhaust grille.

During surgical activity under incorrect behavior conditions, a further increase in air microbial contamination was observed, reaching values of 74, 44, and 93 CFU/m^3^, and 8, 8, and 16 IMA at the operating table, the exhaust grille, and the entrance, respectively.

No fungi were isolated during the C condition, while fungi were isolated by active sampling during NC condition at the operating table (1 CFU/m^3^ of *Penicillium* spp.).

The air sampling performed in the corridor during routine operational conditions yielded 50 CFU/m^3^.

### 3.2. Particle Counting

At the operating table, the number of particles ≥0.5 μm/m^3^ increased from 1,848 P/m^3^ (corresponding to ISO class 5) in the R condition to 64,783 P/m^3^ (corresponding to ISO class 7) during the C condition and to 82,696 P/m^3^ (corresponding to ISO class 7) during the NC condition. Particle air contamination increased by 27.6% during the NC condition compared to the C condition (Table 1).

### 3.3. Microclimatic Measurements

Table 2 shows the median and average values of microclimatic parameters under the R, C, and NC conditions and the different measured points. Standard deviations with respect to the average values are also given.

**Figure 2:**
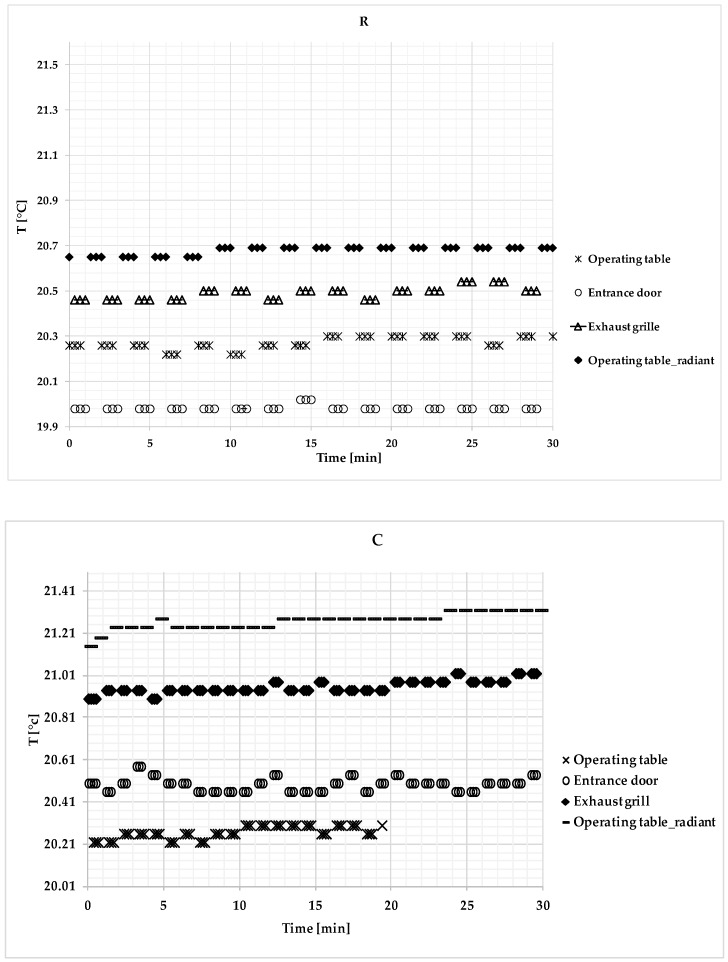

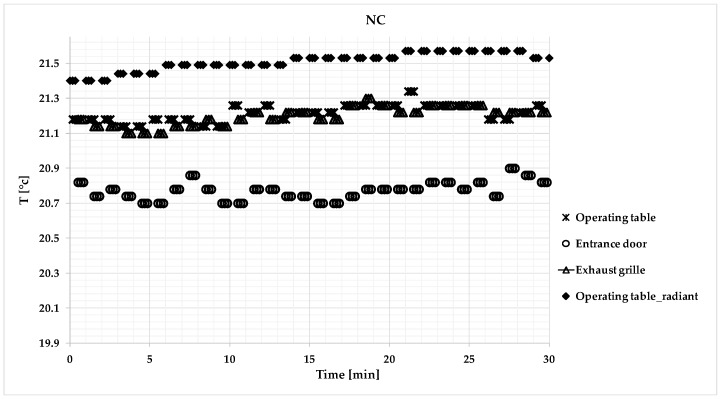
Air temperature values measured during the different operating theatre (OT) conditions (R, C, and NC) at the operating table, exhaust grille, and the entrance door.

**Figure 3:**
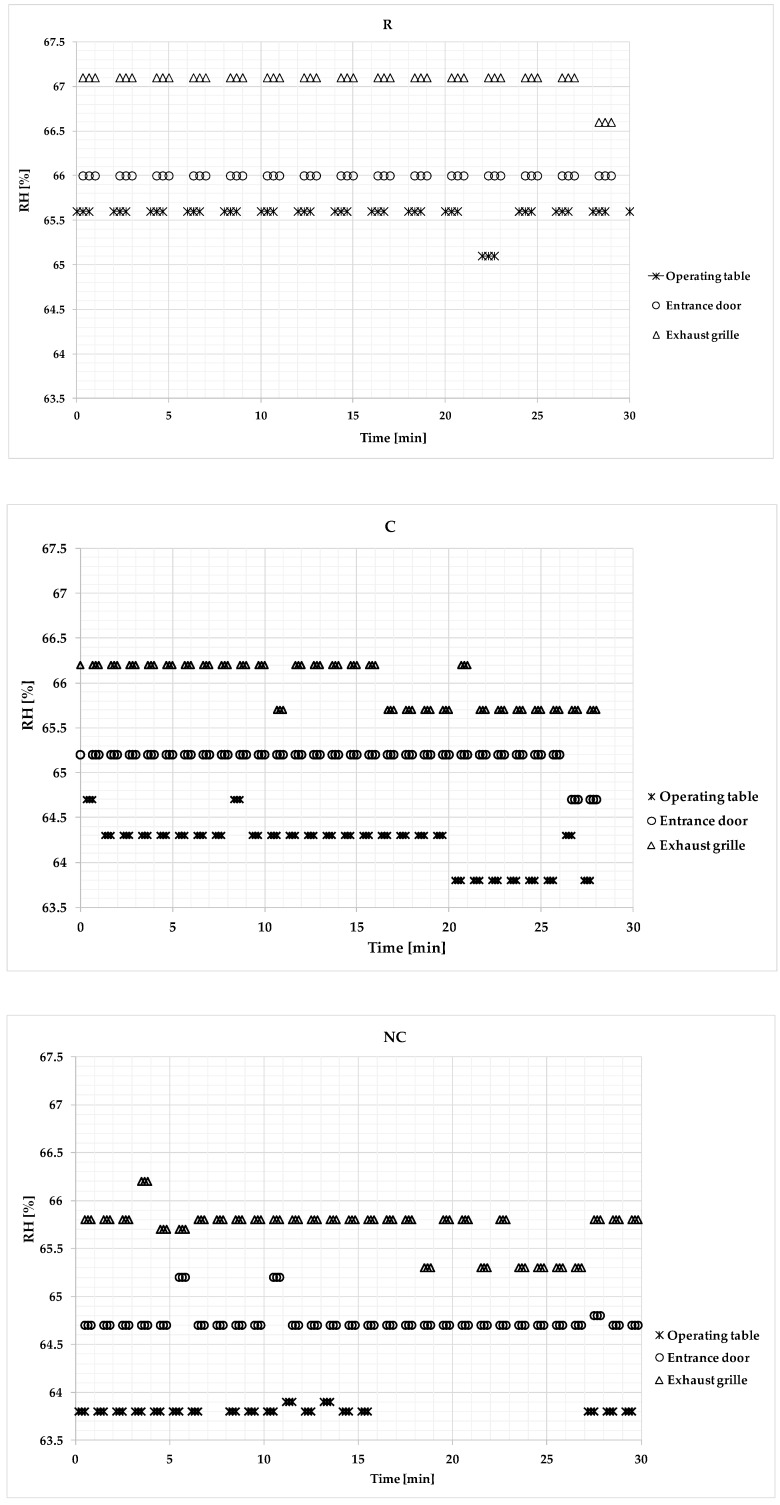
Air relative humidity values measured during different OT conditions (R, C, and NC) at the operating table, exhaust grille, and entrance door.

**Figure 4:**
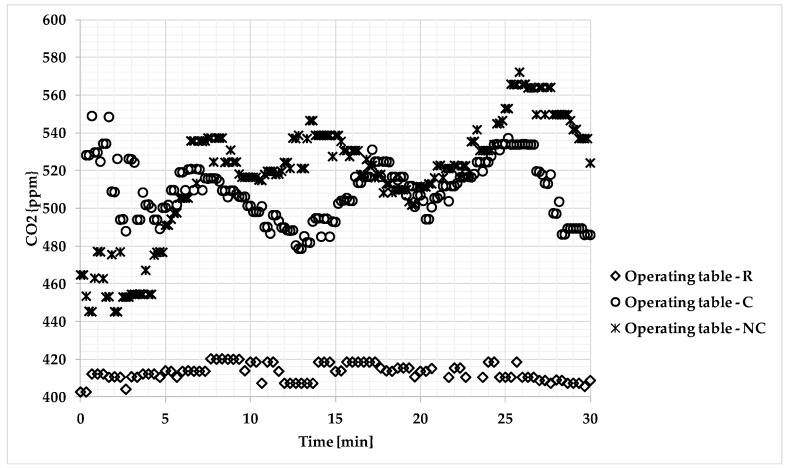
CO_2_ concentration values measured during different OT conditions (R, C, and NC) at the operating table.

Time series of the indoor air temperature, humidity, and CO_2_ concentration values recorded in the different conditions, i.e., R, C, and NC, are given in Figure 2, Figure 3 and Figure 4

Figure 2 shows the air temperature values measured at the operating table, at the exhaust grille, and at the entrance door, including radiant temperature values measured at the operating table.

In particular, during the R condition, the maximum and minimum air temperature values were 20.30 °C and 20.22 °C at the operating table, 20.02 °C and 19.98 °C at the entrance door, and 20.54 °C and 20.46 °C at the exhaust grille. The maximum and minimum radiant temperature values were 20.69 °C and 20.65 °C at the operating table. During the C condition, the maximum and minimum air temperature values were 21.02 °C and 20.82 °C at the operating table, 20.70 °C and 20.46 °C at the entrance door, and 21.02 °C and 20.9 °C at the exhaust grille. The maximum and minimum radiant temperature values were 21.32 °C and 21.24 °C at the operating table. During the NC condition, the maximum and minimum air temperature values were 21.30 °C and 21.10 °C at the operating table, 20.90 °C and 20.71 °C at the entrance door, and 21.30 °C and 21.10 °C at the exhaust grille. The maximum and minimum radiant temperature values were 21.60 °C and 21.41 °C at the operating table.

Figure 3 provides the relative humidity values recorded at the operating table, exhaust grille, and entrance door. The maximum value (67.1%) was recorded at the exhaust grille during the R condition, while the lowest value (63.40%) was recorded at the operating table during the NC condition. The highest values were generally detected at the exhaust grille, while the lowest values were recorded at the operating table.

The CO_2_ concentration values in ppm measured at the operating table for all of the OT conditions are provided in Figure 4. The maximum value (542 ppm) was recorded during the C condition, while the lowest value (409 ppm) was recorded during the R condition.

## 4. Discussion

This study describes an approach of evaluating biological, particle, and microclimatic air quality in conventional operating theatres, which was applied during two simulated hip arthroplasties in two different conditions of surgical staff behavior. The surgical staff behavior had a strong impact on indoor air quality and ventilation efficacy in operating theatres equipped with effective HVAC systems with unidirectional or turbulent flow.

As in other recent studies [22,29,36,37,43], microbial contamination during surgical activity was lower than the recommended values of 180 CFU/m^3^ and 25 IMA [10,14,39]. Pasquarella et al. demonstrated that 80 CFU/m^3^ could be reached during operational conditions [29], and Vonci et al. demonstrated that 50 CFU/m^3^ could be recorded in operating theatres equipped with turbulent flow ventilation at 15 air changes per hour [44]. In a recent revision, Stockwell et al. reported a microbial air contamination of 20 CFU/m^3^ in hospital areas equipped with conventional mechanical ventilation systems [45].

In this study we obtained microbial contamination levels below the recommended values even in the NC condition, showing that currently used turbulent HVAC systems are more efficient than those in the past, as highlighted in previous studies [22,29,37,42,46]; therefore, keeping operational threshold values of 180 CFU/m^3^ and 25 IMA could lead to an underestimation of the risk. In particular, it was shown that some turbulent OTs complied with ISO 5 class [46].

During the surgical operation performed under the C condition, we obtained values of 13 CFU/m^3^ and 2 IMA, which were similar to the threshold values recommended in unidirectional airflow ventilated operating theatres (10 CFU/m^3^ and 2 IMA) [10,12,14], thereby supporting the evidence stating that current HVAC systems are more efficient and can reach the same air quality as that obtained by unidirectional airflow systems under correct surgical behavior conditions. 

The bacteria contamination values (50 CFU/m^3^) recorded in the corridor during routine operational conditions were lower than the values observed during the NC conditions at the operating table and door entrance, which were 74 and 93 CFU/m^3^ respectively. These findings were consistent with the results of a previous study [29] which showed, in some cases, not significant differences between bacterial air contamination in the OT and in the corridor, and even bacterial air contamination higher in the OT than in the corridor in one case.

The particle counting results further confirmed the higher efficiency of currently used turbulent HVAC systems, with similar particle numbers as the recommended levels for unidirectional airflow plants in R conditions and the maintenance of ISO 7-required levels during C and NC conditions for ≥0.5 µm sized particles.

Microbial air contamination, which was measured both by active and passive samplings, and ≥0.5 µm-particle contamination consistently yielded the lowest values under “at rest” conditions and the highest values under “incorrect” conditions.

From a microclimatic point of view, different studies demonstrated the strong influence of total traffic flow and the number of people present in an OT. There was evidence of an important variation in the OT microclimate (i.e., deviation from the standard limit values), which was strictly connected to behavior of surgical teams. In particular, there was a strong relationship between the ventilation system, its air flow scheme, and staff behavior, and the air motion and air temperature field in high-performance hospital OTs equipped with HVAC systems with unidirectional and turbulent flow. Therefore, the need for training and control of surgeon/medical and nursing staff should be emphasized [25,28,32,35,47].

In this study, the local increase of air motion and the turbulence effects induced by staff presence under the C condition was evident. When comparing trends for the R and C conditions, the velocity values recorded at the operating table were different and the air motion was increased by staff presence during the C condition; a further increment during the NC condition was observed. At the entrance door, a reduction in the air velocity from the R condition to the C condition was followed by air motion stabilization during the NC condition. These effects were typical of a turbulent motion mainly characterized by flow field irregularity of the main variables and diffusivity, i.e., irregularities due to a rapid mixing of fluid portions. The turbulent motion determined a certain dispersion of the air velocity values in the studied conditions, namely, R, C, and NC, as deduced from the Table 2.

The air temperature showed the lowest mean values at the entrance door due to the turbulent effects. The data dispersion at the operating table was influenced by surgical staff presence and behavior. The average radiant temperature values at the operating table were consistently higher than those achieved by the thermohygrometers. The highest air temperature values were recorded at the operating table, which was clearly due to surgical staff presence and movements, as it was also confirmed by the highest values of the air radiant temperature at the same sampling point. The sliding door opening/closing phases did not seem to have a substantial impact on the temperature. The temperature variations over time were low. From comparisons of the three conditions (R, C, and NC), the variation of the air temperature had a similar trend over time, thereby proving that the turbulent flow ventilation scheme immediately affected the whole environment.

The relative air humidity variations over time were low, corresponding to average values in the range of 63%–68% for all of the different OT conditions that were studied.

CO_2_ concentration levels in the OT were affected by the presence of the surgical team for the C condition, mainly by the opening/closing door and incorrect behavior of the surgical team (NC condition). CO_2_ values observed during the C condition showed a fluctuating trend, but they continued to increase over time in the NC condition. 

The dispersion of CO_2_ concentration measurements appeared high. The difference was clearer for the data acquired for the C and NC conditions, when the medical staff remained inside the room and opening/closing of the door occurred. The standard deviations for the C and NC conditions were 3 and 6 times that computed for the R condition, respectively. 

The obtained results were also in accordance with evidence in the literature, demonstrating that air flow patterns and air velocity and temperature distributions were disrupted by the amount, behavior, and upright and bending positions of surgical staff [20,48].

The microclimatic parameters agreed with current recommendations [10,15,49,50], although variations during the NC condition were observed.

## 5. Conclusions

Our study shows the negative influence of the surgical team’s incorrect behavior on operating theatre microbial and particle air contamination and microclimatic parameters. The microbial contamination values were much lower than the current recommended threshold values for operational conventionally ventilated operating theatres during simulated hip arthroplasties with the surgical team behaving incorrectly. This highlights the need for a revision of these threshold values. During operations where the surgical team behaved correctly, very low microbial contamination was detected, with results not too far from those recommended for the unidirectional air flow plant system. 

This contribution is important considering the wider use of conventional operating theatres for hip arthroplasties, particularly in light of the ongoing debate regarding unidirectional air flow ventilating systems as a risk factor for surgical site infections in hip arthroplasties. To our knowledge, this study is the first to evaluate air quality in OTs including both sampling methods (i.e., active and passive) and microclimate monitoring. The obtained results represent a useful basis for further simulated interventions, during which the modification of particle and microbiological contamination and variations in the microclimatic parameters influenced by incorrect behavior could be thoroughly assessed. The use of this approach for real hip arthroplasties with a wider collection of comparable data will provide important knowledge regarding the air quality in current conventional operating theatres, potentially leading to a revision of the threshold values. 

Whichever HVAC system is installed, it is essential to guarantee operating theatre air quality. Poor management of the HVAC or incorrect operator behavior could undermine this economic investment and expose patients to the risk of surgical site infections. In this regard, air microbiological monitoring can be a useful tool to assess air quality, test the effectiveness of preventive measures, and identify hazardous situations.

## Figures and Tables

**Table 1:** Microbial air contamination values obtained via active (CFU/m^3^) and passive (IMA) sampling, and particle (≥0.5 µm) contamination at different sampling point during rest, correct, and not correct conditions.

	Operating Table			Exhaust Grille			Entrance Door		
	R	C	NC	R	C	NC	R	C	NC
**CFU** **/** **m^3^**	2	13	74	3	23	44	4	29	93
**I** **MA**	0	2	8	0	6	8	2	4	16
**Particles ≥ 0.5 µm**	1848	64,783	82,696	-	-	-	-	-	-

R = at rest; C = correct conditions; NC = not correct conditions; CFU = colony-forming units; IMA = Index of Microbial Air contamination.

**Table 2:** Median values, averages, and standard deviation values of the microclimatic parameters.

	Air velocity [m/s]		Temperature [°C]				Relative Humidity [%]			CO_2_ [ppm]	
	OPERATING TABLE	Entrance Door	Operating Table Radiant	Operating Table	Exhaust Grille	Entrance Door	Operating Table	Exhaust Grille	Entrance Door	Operating Table	
**Median**	0.023	0.135	20.69	20.26	20.50	19.98	65.60	67.10	66.00	410.39	**At rest**
**Average**	0.031	0.134	20.68	20.27	20.49	19.98	65.57	67.05	66.00	411.99	
**Standard deviation**	0.04	0.02	0.02	0.03	0.03	0.01	0.12	0.16	0.10	0.18	
**Median**	0.051	0.093	21.28	20.94	20.98	20.50	64.30	66.20	65.20	505.91	**Correct condition**
**Average**	0.069	0.095	21.27	20.93	20.97	20.51	64.21	65.98	65.16	506.31	
**Standard deviation**	0.06	0.05	0.03	0.06	0.03	0.05	0.26	0.25	0.13	0.52	
**Median**	0.19	0.092	21.53	21.22	21.21	20.78	63.80	65.80	64.71	522.50	**Not correct condition**
**Average**	0.15	0.11	21.50	21.20	21.20	20.80	63.60	65.70	64.70	520.52	
**Standard deviation**	0.07	0.07	0.05	0.05	0.05	0.05	0.21	0.22	0.12	0.86	
